# Identification of Anziaic Acid, a Lichen Depside from *Hypotrachyna* sp., as a New Topoisomerase Poison Inhibitor

**DOI:** 10.1371/journal.pone.0060770

**Published:** 2013-04-08

**Authors:** Bokun Cheng, Shugeng Cao, Victor Vasquez, Thirunavukkarasu Annamalai, Giselle Tamayo-Castillo, Jon Clardy, Yuk-Ching Tse-Dinh

**Affiliations:** 1 Department of Biochemistry and Molecular Biology, New York Medical College, Valhalla, New York, United States of America; 2 Department of Biological Chemistry and Molecular Pharmacology, Harvard Medical School, Boston, Massachusetts, United States of America; 3 Unidad Estrategica de Bioprospeccion, Instituto Nacional de Biodiversidad (INBio) and Escuela de Química, Universidad de Costa Rica, San José, Costa Rica; 4 Department of Chemistry and Biochemistry, Florida International University, Miami, Florida, United States of America; Albert-Ludwigs-University, Germany

## Abstract

Topoisomerase inhibitors are effective for antibacterial and anticancer therapy because they can lead to the accumulation of the intermediate DNA cleavage complex formed by the topoisomerase enzymes, which trigger cell death. Here we report the application of a novel enzyme-based high-throughput screening assay to identify natural product extracts that can lead to increased accumulation of the DNA cleavage complex formed by recombinant *Yersinia pestis* topoisomerase I as part of a larger effort to identify new antibacterial compounds. Further characterization and fractionation of the screening positives from the primary assay led to the discovery of a depside, anziaic acid, from the lichen *Hypotrachyna* sp. as an inhibitor for both *Y. pestis* and *Escherichia coli* topoisomerase I. In *in vitro* assays, anziaic acid exhibits antibacterial activity against *Bacillus subtilis* and a membrane permeable strain of *E. coli*. Anziaic acid was also found to act as an inhibitor of human topoisomerase II but had little effect on human topoisomerase I. This is the first report of a depside with activity as a topoisomerase poison inhibitor and demonstrates the potential of this class of natural products as a source for new antibacterial and anticancer compounds.

## Introduction

DNA topoisomerases are ubiquitous enzymes that play essential roles in controlling the topological state of DNA to facilitate and remove barriers for vital cellular functions including DNA replication, transcription, recombination and repair [1], [2]. Topoisomerase enzymes utilize an active site tyrosine side chain for nucleophilic attack of the DNA phosphodiester linkage, resulting in the formation of a covalent reaction intermediate with the tyrosine covalently linked to the phosphoryl end of the cleaved DNA. Topoisomerase poison inhibitors can promote cell death by increasing the accumulation of this covalent protein-DNA intermediate [3]. These inhibitors can therefore be highly effective antibacterial and anticancer agents. A number of topoisomerase poison inhibitors have been isolated as natural products. These include camptothecin which targets type IB human topoisomerase, and has been followed by the development of more soluble analogs that are useful in clinical treatment of different types of cancers [4]. Quinolones are highly effective synthetic molecules that act as poison inhibitors targeting type IIA bacterial topoisomerases [5].

New antibacterial agents acting on a novel target are very much needed to combat the serious global health problem of bacterial pathogens resistant to quinolones and other existing antibacterial therapeutics [6], [7]. Every bacterium has at least one type IA DNA topoisomerase that could potentially be targeted by a new class of topoisomerase poison [8]. Recombinant bacterial topoisomerase I with a mutation mimicking the effect of topoisomerase poison has been used to demonstrate that the accumulation of topoisomerase I covalent cleavage intermediate indeed leads to bacterial cell death [9]–[11]. The cell death mechanism involves reactive oxygen species [12], as demonstrated for quinolones and other bactericidal antibiotics [13], [14]. A target based high throughput screening (HTS) assay was developed for bacterial topoisomerase I to identify agents that can lead to higher levels of DNA cleavage product. *Yersinia pestis* can potentially be used as a bioweapon so the HTS assay was optimized for *Y. pestis* topoisomerase I. Pilot screening was carried out against collections of small molecules and natural product extracts. Great diversity of molecular structures can be found among natural products and antibacterial activities can often be discovered in natural products from various sources [15]. Follow up experiments for one of the natural product screening hits led to the identification of the depside anziaic acid as the active compound. Our results show that a depside can act as a topoisomerase poison, suggesting that this class of natural product could be exploited to provide novel leads for antimicrobial and anticancer agents.

## Materials and Methods

### Topoisomerase Enzymes

Recombinant *Y. pestis* and *E. coli* DNA topoisomerase I were purified as previously described [9], [16]. Human topoisomerase I and II were purchased from TopoGen. *E. coli* DNA gyrase was purchased from New England BioLabs.

### HTS Assays

Oligonucleotide substrate (5′-CTTATGCAATGCGCT↓TTGGGCAAACCAAGAGAGCATAAG-3′) with CAL Fluor Red 610 fluorophore at the 5′-end and BHQ-2 quencher at the 3′-end was custom synthesized by Biosearch Technologies. This oligonucleotide forms a stem-loop structure with a single cleavage site for *Y. pestis* and *E. coli* topoisomerase I in the single-stranded loop region indicated by the arrow. Ten microliter aliquots of a premix of 100 nM oligo substrate, 100 nM *Y. pestis* topoisomerase I in 10 mM Tris-HCl, pH 8, 0.5 mM MgCl_2_ were dispensed into 384-well Corning 3821 low volume plates followed by pin transfers of 33 nL of library compounds at the NERCE/NSRB HTS facility. DMSO was used as negative control at 0.33% while compound NSC28086 was used as a positive control at 0.25 mM concentration. After 60 min at room temperature, fluorescence (Ex/Em wavelengths of 590/610 nm) was recorded with the Envision3 plate reader. The assay was carried out in duplicates for 7,105 small molecules and 2,816 natural product extracts at NERCE/NSRB.

### Natural Product Library

The natural product libraries, which were prepared in Costa Rica (collection permits 307-2003-OFAU, R-CM-03-2006, R-CMINBio-06-2006, R-CM-INBio-082-2009, R-CM-INBio-04-2009, R-CM-INBio-088-2009 and R-CM-INBio-094-2010), consisted mainly of pre-fractionated extracts from microbial sources, such as fungal endophytes and marine bacteria, although extracts from other sources such as marine invertebrates, cyanobacteria and lichens were also included [17]. Extracts were suspended in dimethyl sulfoxide (DMSO) at a concentration of ∼15 mg/mL. The compound libraries were stored at −20°C in desiccated storage containers. Screening of the libraries ICBG16 (1408), NCDDG7 (1056) and NCDDG8 (352) yielded two confirmed hits, PL2050/C13 and PL2050/D12. Both hits were originated from the same lichen sample, *Hypotrachyna* sp.

### Compound Isolation and Identification

#### Lichen sample collection and extraction

Approximately 5.25 g of the lichen *Hypotrachyna* sp. (Lecanorales, Parmeliaceae) collected at Quetzales National Park, Central Pacific, Costa Rica (Permit R-CM-INBio-30-2007-OT) on February 2009, afforded 4.53 g of dry material upon drying. The dry powder was extracted with 100 mL 95% ethanol in an ultrasonic bath for 20 minutes and repeated 3 times, to yield 807.4 mg of crude extract.

#### Isolation and identification

**Figure 1:**
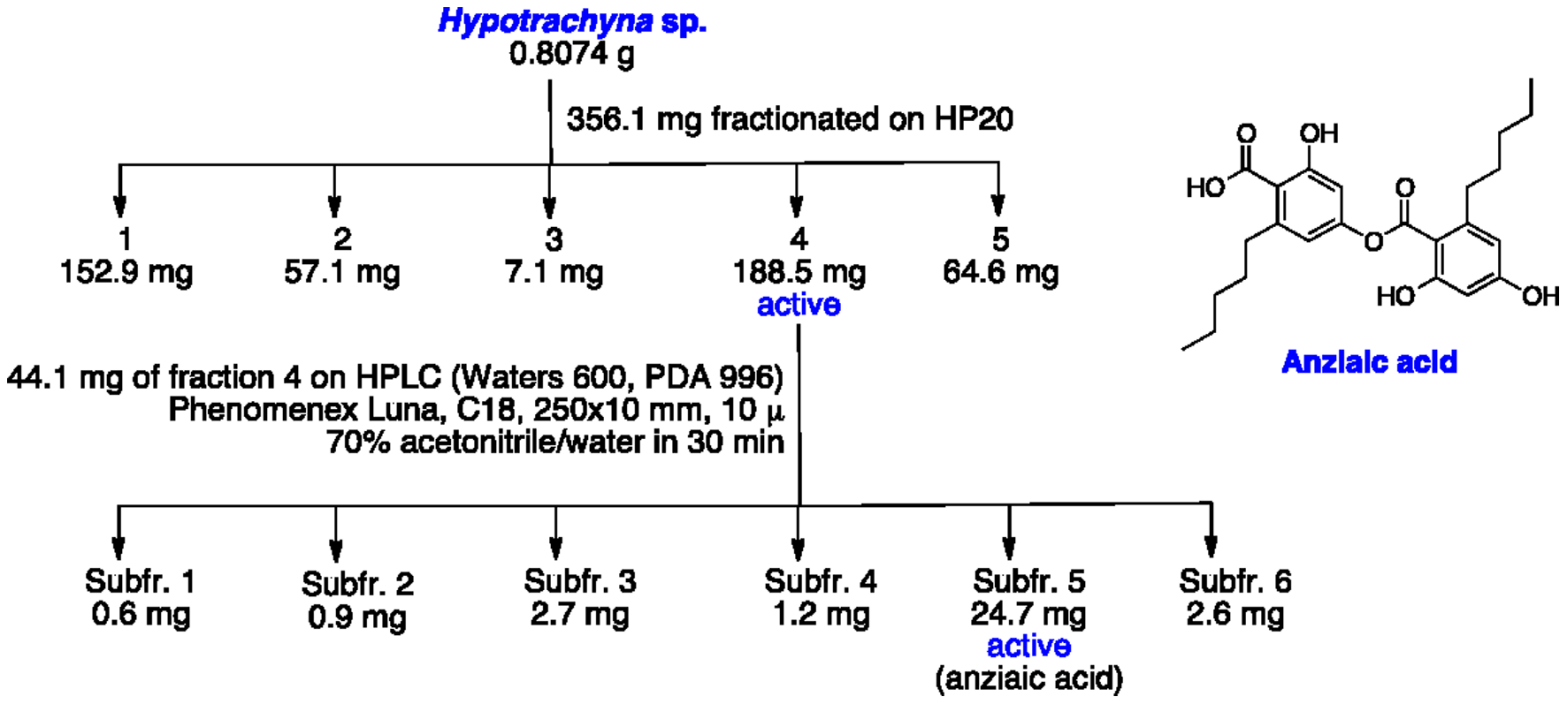
Isolation tree of anziaic acid.

A solid phase extraction method was employed (Figure 1). Approximately 356.1 mg of crude extract of *Hypotrachyna* adsorbed in Diaion HP-20ss (3∶1) were loaded on a column containing 4 g of Diaion HP-20ss and eluted with 15 mL each of water:ethanol 8∶2, 1∶1, 2∶8, ethanol and isopropanol:dichloromethane 8∶2 to afford five fractions. Fraction 4 afforded 188.5 mg of SScode 86608. A HPLC separation (HPLC Waters 600 with a Waters detector PDA 996) was conducted on fraction 4 (44.1 mg) using isocratic conditions (70% acetonitrile:water for 30 min; Phenomenex Luna, C18, 250 X 10 mm, 10 micron) to obtain subfraction 5, SScode 86610, (24.7 mg, *T_R_* 18 min). SScode 86610 was identified as anziaic acid by comparison of NMR (Figure S1, S2, Table S1) and mass spectroscopy (Figure S3) data with literature values [18].

### Topoisomerase Activity Assays

Inhibition of relaxation activity of 10 ng of *Y. pestis* and *E. coli* topoisomerase I were assayed with 250 ng of supercoiled pBAD/Thio plasmid DNA substrate purified by CsCl gradient. The relaxation reaction was carried out in 20 µL of 10 mM Tris-HCl, pH 8.0, 50 mM NaCl, 0.1 mg/mL gelatin with 0.5 mM MgCl_2._ A higher concentration of 6 mM MgCl_2_ was used in some experiments. Tween 20, when present was at 0.0025%. After 30 min at 37°C, the reactions were terminated and analyzed by agarose gel electrophoresis as previously described [16], [19].

The effect of inhibitors on the accumulation of DNA cleavage product for *E. coli* topoisomerase I was assayed with 5′-^32^P-labeled 216 base long single-stranded DNA generated by denaturation of a PCR product [20]. The labeled DNA (50 ng) was incubated with 100 ng of *E. coli* topoisomerase I in 5 µL of 10 mM Tris-HCl, pH 8.0, 2 mM MgCl_2_ at 37°C for 30 min to allow the establishment of a DNA cleavage-religation equilibrium. The reaction was terminated by the addition of an equal volume of sequencing gel loading buffer. The level of DNA cleavage products was analyzed by electrophoresis in a 7% sequencing gel followed by Phosphor-Imager analysis of the dried gel.

Human topoisomerase I and IIα (from TopoGen) relaxation assays and *E. coli* DNA gyrase (from New England BioLab) supercoiling assay were carried out as recommended by the suppliers. Relaxed plasmid DNA substrate for DNA gyrase was purchased from New England BioLabs. Human topoisomerase I and II DNA cleavage assays were carried out as described previously [19]. For gyrase DNA cleavage assay, enzyme-DNA reactions were treated with 1% SDS for 5 min followed by proteinase K (0.5 µg/mL) digestion for 30 min before analysis in agarose gel in the presence of ethidium bromide.

### Antibacterial Assay

Inhibition of bacterial growth by natural product extracts was measured initially in 96 well plates with lid in a Perkin Elmer HTS7000 Plus plate reader with brief shaking at 20 min intervals at 37°C. IC_50_s were defined as concentrations that inhibited growth by 50% after 24 h as determined by absorption readings at 600 nm.

MICs of isolated inhibitor against different bacterial strains grown in cation-adjusted Mueller- Hinton Broth were measured with standard microdilution procedures. Complete growth inhibition was recorded after 24 h in a 37°C incubator.

### Mammalian Cell Cytotoxicity Assay

Human pulmonary artery endothelial cells (HPAEC) were obtained from Cell Applications, Inc. (San Diego, CA, USA) and cultured in endothelial cell media supplemented with subculture Reagent kit. Approximately 5000 cells in 100 µL were placed in each well of a 96-well plate. After the addition of an inhibitor or DMSO, cells were incubated overnight before assay for viability with the Promega CellTiter-Glo Luminescent Cell Viability Assay according to the manufacturer’s protocol.

## Results

### Validation of HTS Assay

The fluorescence emission of the fluorophore at the 5′-end the oligonucleotide substrate is quenched by the quencher at the 3′-end. Shifting of the topoisomerase I cleavage-religation equilibrium towards DNA cleavage is expected to lead to an increase in fluorescence emission. This was validated using the G116S mutant form of *E. coli* topoisomerase I previously shown to be defective in DNA religation [9]. Addition of this mutant enzyme resulted in >5 fold increase in fluorescence signal over the wild-type enzyme when fluorescence was measured with a BioTek Synergy HT plate reader. A small molecule that can be used as positive control compound NSC28086, was identified from a separate set of analysis carried out on the NCI Diversity Set of compounds to inhibit relaxation activity of *E. coli* and *Y. pestis* topoisomerase I, and increase level of the cleavage product (results to be published). This compound can also result in >5 fold increase in fluorescence signal over DMSO control when present at 0.25 mM concentration. Screening parameters were optimized at the NERCE/NSRB screening center to achieve a Z’ factor of 0.7–0.8 on different days.

### Pilot Screening of Small Molecule and Natural Product Library

Pilot screening was carried out in duplicate for 7,105 small molecules and 2,816 natural product extracts at NERCE/NSRB. A column of negative control (DMSO) was included in each plate. The signal from each compound well was divided by the average of the negative control signal from the same plate. Compounds that resulted in ratio of >1.6 in both duplicated assays were selected as hits corresponding to a hit rate of 0.3% after elimination of compounds with high intrinsic fluorescence in counter assay. The thirty selected hits included ten natural product extracts.

### Secondary Assay

**Figure 2:**
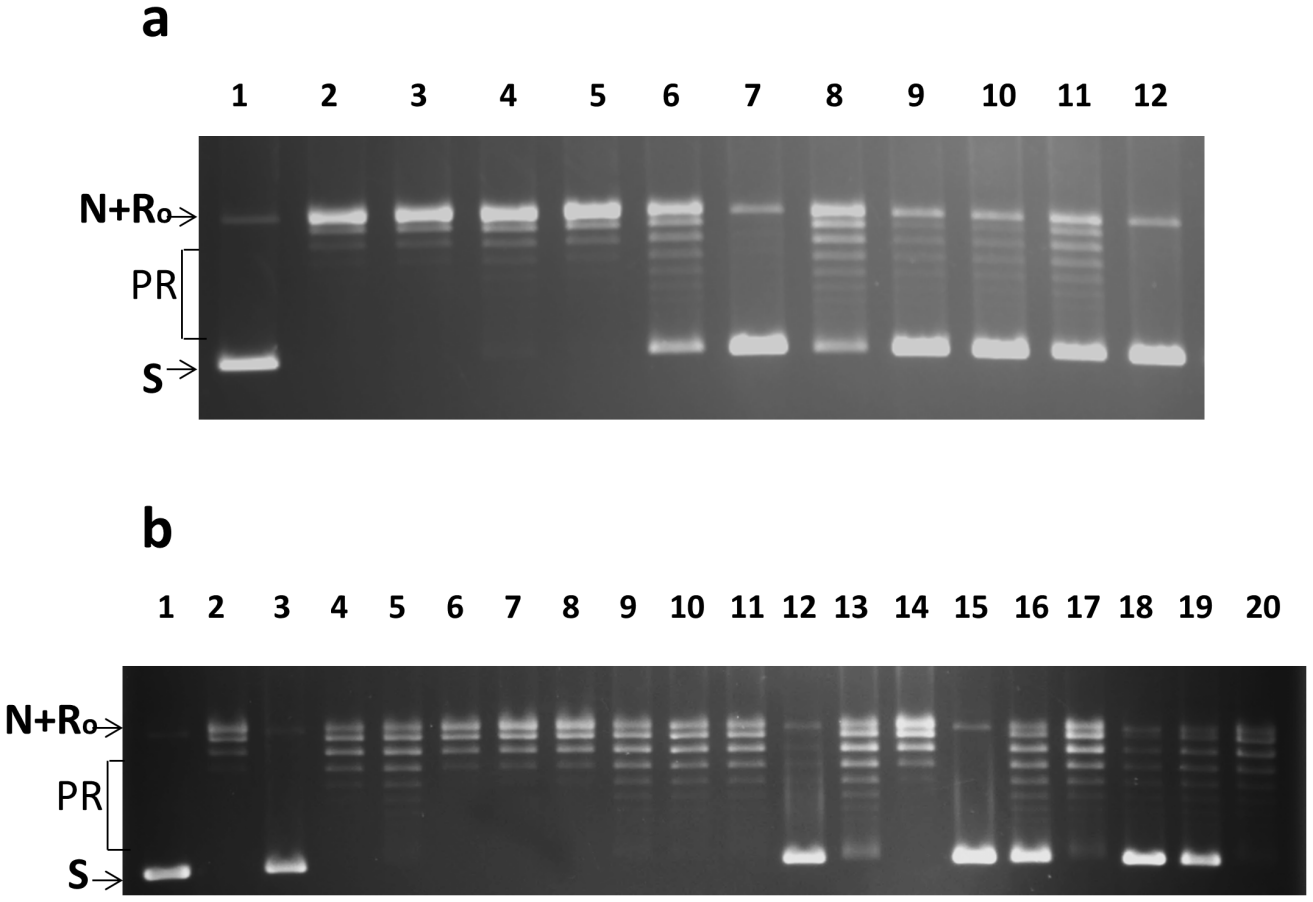
Inhibition of *E. coli* topoisomerase I relaxation activity by lichen extracts and fractions. (a) Secondary assay of natural product hits from HTS using negatively supercoiled DNA plasmid substrate. Lane 1: no enzyme. Relaxation activity of 10 ng of *E. coli* topoisomerase I was assayed in the presence of DMSO (lane 2) or 80 µg/mL of natural product extracts hits (lane 3–12). Extracts PL2050/C13 and PL2050/D12 prepared from lichen *Hypotrachyna sp*. samples were present in lanes 7 and 8 respectively. (b) Assay of HP20ss fractionated extracts of lichen *Hypotrachynasp.* samples. Lane 1: no enzyme control. Lane 2: Enzyme with DMSO control. Lanes 3–20: serial 4-fold dilutions (12, 3, 0.75 µg/mL) of unfractionated total extract (lanes 3–5), Fraction 1 (lanes 6–8), Fraction 2 (lanes 9–11), Fraction 3 (lanes 12–14), Fraction 4 (lanes 15–17), Fraction 5 (lanes 18–20). S: supercoiled plasmid DNA substrate. N: nicked plasmid DNA. Ro: Relaxed closed plasmid DNA. PR: partially relaxed plasmid DNA.

One microliter of 15 mg/mL selected natural product extract hits was available for confirmation of the primary assay result using the BioTek HT plate reader at New York Medical College. From the 10 natural product extracts among the primary assay hits, extracts PL2050/C13 and PL2050/D12 were found to result in a fluorescence signal ratio of 3.6 and 1.5 respectively at 50 µg/mL concentration when compared to DMSO control. In the secondary assay, these extracts were tested for inhibition of the relaxation activity of *E. coli* topoisomerase I at 80 µg/mL. Complete inhibition of relaxation activity was observed for PL2050/C13 and partial inhibition was seen for PL2050/D12 (Figure 2a). Both of these confirmed hits were prepared from lichen *Hypotrachyna sp*. samples. Extract of this lichen was prioritized for further studies over the other extracts that also showed topoisomerase inhibition.

### Identification of Bacterial Topoisomerase I Poison Inhibitor with Antibacterial Activity

**Figure 3:**
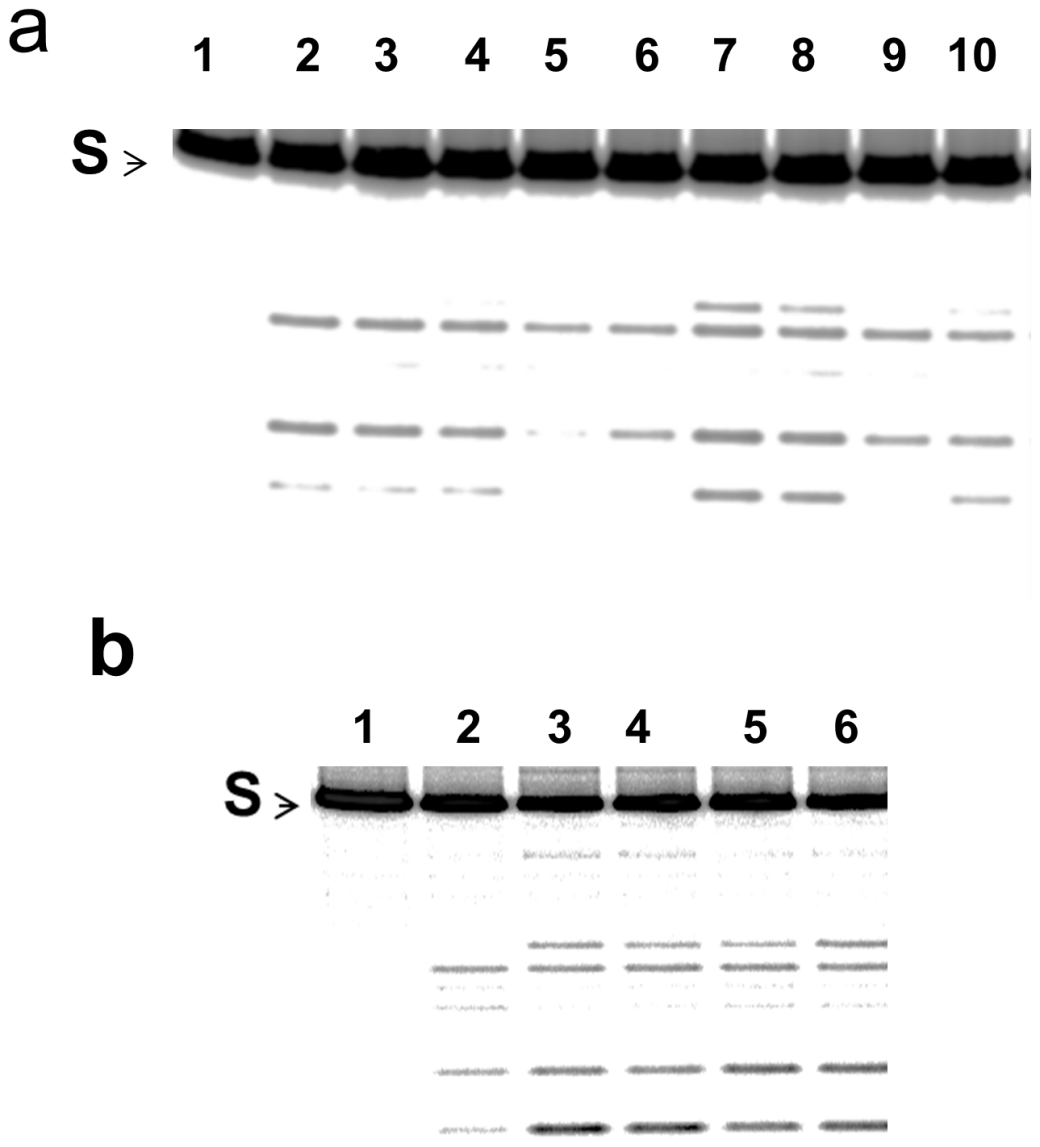
Effect of lichen *Hypotrachyna sp*. HP20ss fractions and anziaic acid on cleavage product accumulation by *E. coli* topoisomerase I. Single-stranded DNA substrate labeled at the 5′-end with ^32^P (S) was incubated with enzyme in the presence of 2 mM MgCl_2_. No enzyme (lane 1), enzyme with DMSO (lane 2). **a.** Unfractionated total extract (lanes 3, 4), fraction 3 (lanes 5, 6), fraction 4 (lanes 7, 8), fraction 5 (lanes 9, 10) with the extract or fraction compounds present at 3.1 µg/mL (lanes 3, 5, 7, 9) or 12.5 µg/mL (lanes 4, 6, 8, 10). **b.** Anziaic acid was present at 0.9 µM (lanes 3), 1.8 µM (lanes 4), 3.7 µM (lanes 5) and 7.2 µM (lanes 6).

Lichen Hypotrachyna sp. extract was subject to chromatography fractionation. After HP20ss fractionation, fractions 3–5 were found to inhibit the relaxation activity of *E. coli* topoisomerase I, with fraction 4 being the most potent (Figure 2b). Antibacterial activity was also monitored with permeable *E. coli* strain BAS3023 with the *imp4213* mutation conferring permeability to small molecules [21], [22] and was found in fractions 3–5 (Table 1). Inhibitors acting with a topoisomerase poison inhibitor mechanism are of greater interest. These can be identified from the effect of the inhibitors on the level of DNA cleavage products formed by bacterial topoisomerase I enzyme from 5′-^32^P labeled single-stranded DNA. The results in Figure 3a showed that only fraction 4 resulted in increased accumulation of DNA cleavage product formed by *E. coli* topoisomerase I as expected from a topoisomerase poison inhibitor. Decrease in cleavage product was seen in the presence of materials from fraction 3 and 5 (Figure 3a). Compounds present in fractions 3 and 5 likely inhibit the relaxation activity by inhibition of the DNA binding or cleavage steps. Further separation of material in Fraction 4 led to the identification of anziaic acid as the active compound (Figure 3b). Anziaic acid exhibited antibacterial activity against *Bacillus subtilis* (ATCC 6633) with an MIC of 6 µg/mL (14 µM), and *E. coli* strain BAS3023 (MIC = 12 µg/mL or 28 µM), but not for *E. coli* strain BW27784 without permeability mutation.

**Table 1:** Growth inhibition of *E. coli* strain BAS3023 by lichen *Hypotrachyna sp*. HP20ss fractions.

Fraction	IC_50_ Growth inhibition (µg/mL)
Total extract	12.5
Fraction 1	negative
Fraction 2	negative
Fraction 3	25
Fraction 4	5
Fraction 5	5

### Effect of Detergent and Mg^2+^ Concentration on Inhibitory Activity

**Figure 4:**
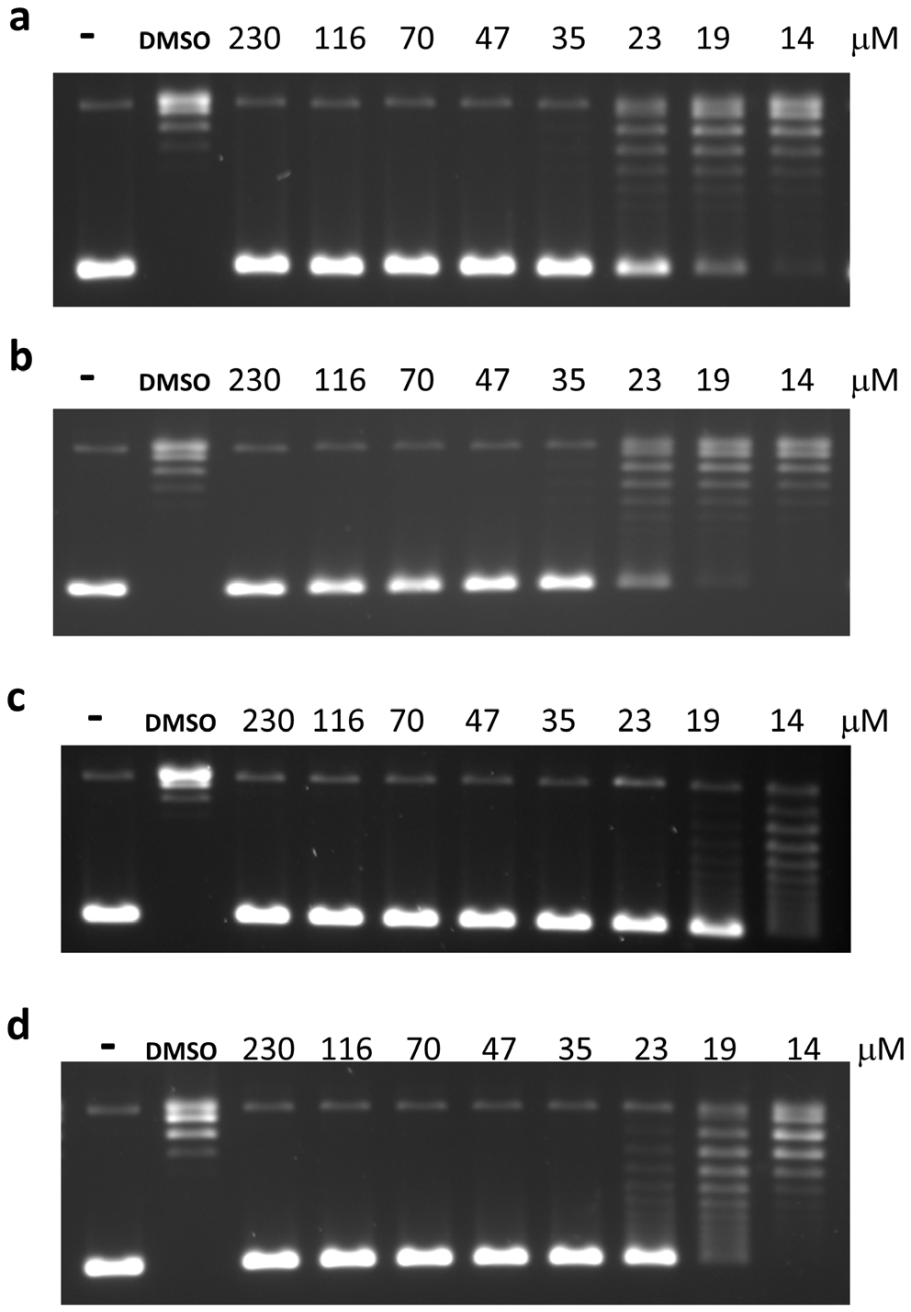
Inhibition of Relaxation activity of *E. coli* topoisomerase I by anziaic acid. Assay was carried out in the presence of DMSO or the indicated concentration of anziaic acid with (a) 0.5 mM MgCl_2_, (b) 0.5 mM MgCl_2_+0.0025% Tween 20, (c) 6 mM MgCl_2_, (d) 6 mM MgCl_2_+0.0025% Tween 20. -: no enzyme control.

**Figure 5:**
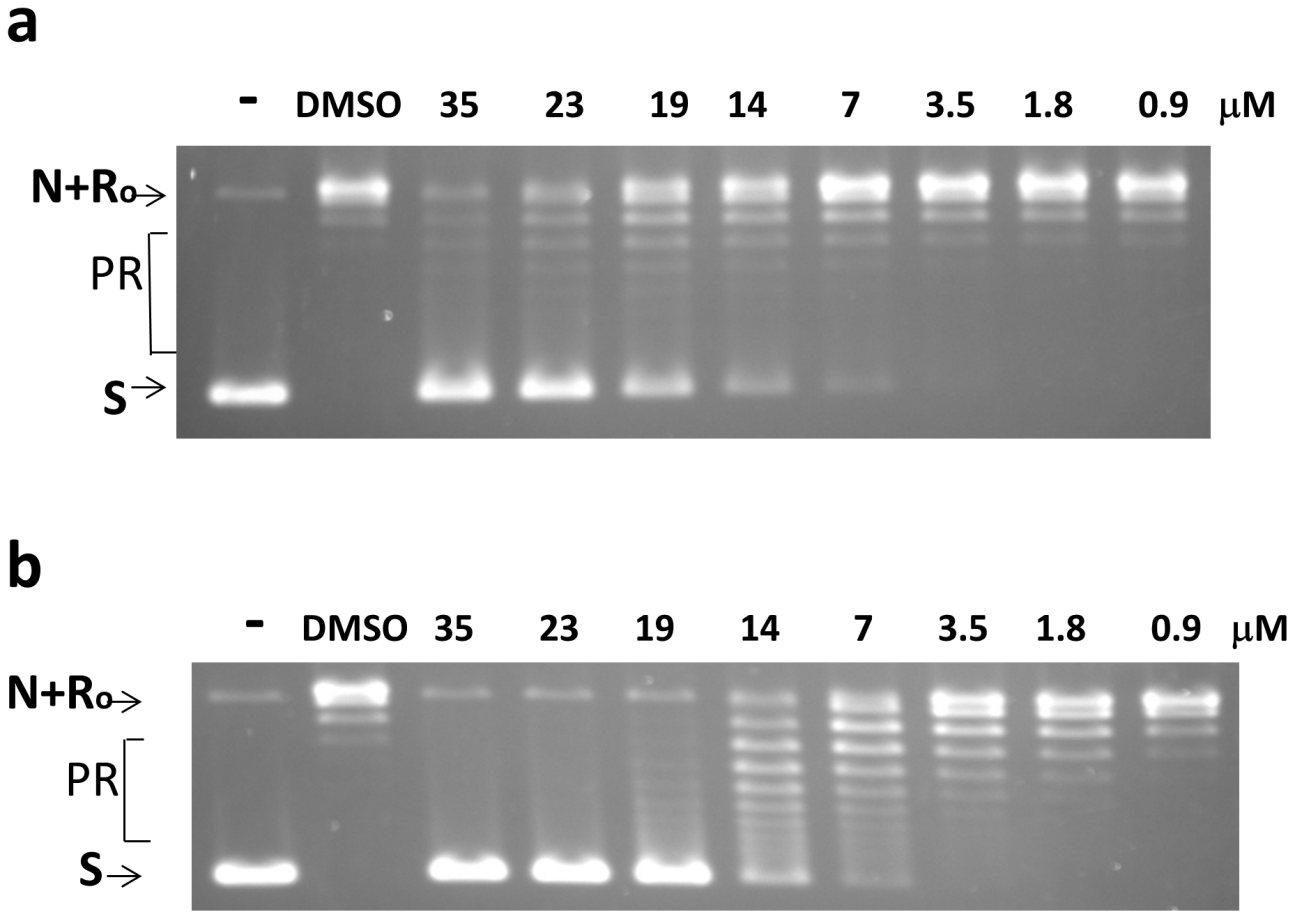
Inhibition of Relaxation activity of *Y. pestis* topoisomerase I by anziaic acid in the presence of different concentrations of Mg^2+^. Assays were carried out in the presence of 0.0025% Tween 20 and MgCl_2_ concentration of 0.5 mM (a) or 6 mM (b). -: no enzyme control. Anziaic acid is present at the concentration indicated.

The presence of 0.0025% Tween 20 had only a minor effect on the inhibition of the *E. coli* topoisomerase I relaxation activity, indicating that anziaic acid is not acting as a promiscuous inhibitor (Figure 4). The structure of anziaic acid suggests that it may act as a chelator of Mg**^2+^**. Divalent ions are required for the religation step and the relaxation activity of bacterial topoisomerase I [23], [24]. However, there is no increase in IC_50_ for relaxation inhibition when Mg**^2+^** concentration was shifted from 0.5 to 6 mM (Figure 4). The inhibition by anziaic acid was more effective at the higher Mg**^2+^** concentration (IC_50_ = 19 µM in the presence of Tween 20). These mechanistic characteristics are also true for the inhibition of relaxation activity of *Y. pestis* topoisomerase I by anziaic acid with IC_50_ = 14 µM in the presence of Tween 20 and 6 mM Mg**^2+^** (Figure 5). The increase in intermediate cleavage product accumulation by *E. coli* topoisomerase I can be seen at 2 mM Mg**^2+^** concentration that is far higher than the concentration of anziaic acid (0.9–7.2 µM) present in the DNA cleavage assay (Figure 3b). It can thus be concluded that inhibition did not result from chelation of free Mg^2+^ by anziaic acid. However, the results did not rule out interference of divalent ions bound at the active site of bacterial topoisomerase I by anziaic acid as the mechanism of inhibition. The concentration of anziaic acid sufficient for increase in DNA cleavage product accumulation (Figure 3b) is >20 fold lower than the IC_50_ for relaxation of the overall catalytic cycle (Figure 4). This is consistent with anziaic acid acting as a poison inhibitor.

### Assays for Inhibition of other DNA Topoisomerases

**Figure 6:**
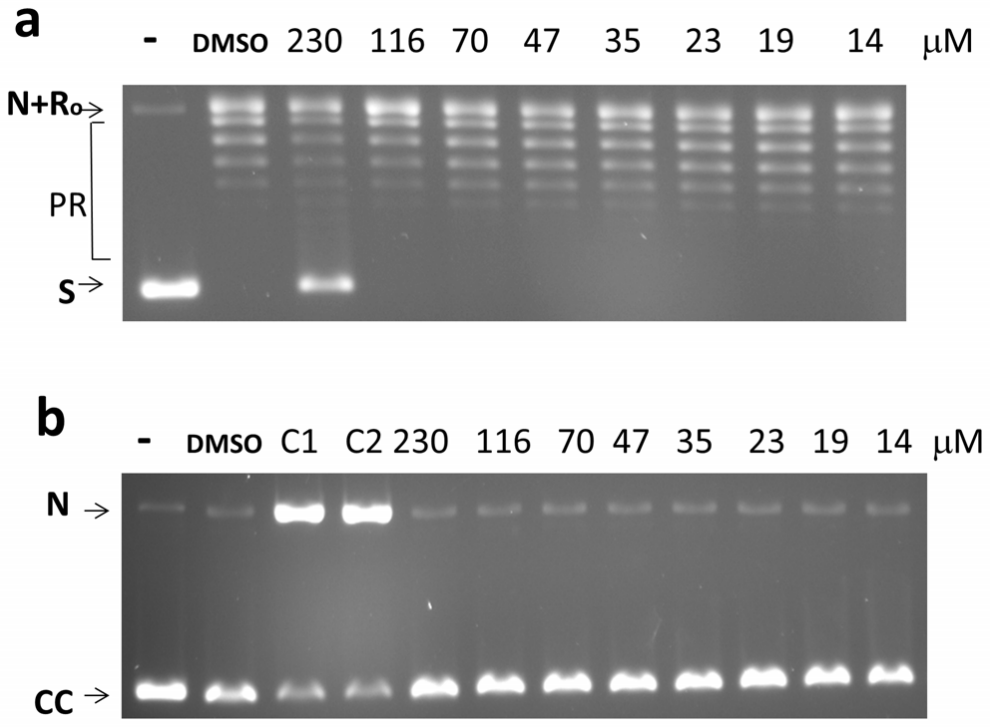
Effect of anziaic acid on type IB human topoisomerase I activity. (a) Assay of relaxation activity –1 U of enzyme was used in each relaxation reaction with 250 ng of supercoiled plasmid DNA. (b) DNA cleavage assay with 5 U of enzyme. Gel electrophoresis was carried out in the presence of 0.5 µg/mL ethidium bromide and camptothecin was used as a positive control at 62.5 µM (C1) or 125 µM (C2). -: no enzyme. S: supercoiled plasmid DN substrate. N: nicked plasmid DNA. Ro: Relaxed closed plasmid DNA. PR: partially relaxed plasmid DNA. CC: Covalently closed circular DNA.

To further evaluate the specificity of topoisomerase inhibition by anziaic acid, the effect on type IB human topoisomerase I was evaluated. The IC_50_ for relaxation inhibition was found to be >10 fold higher than the IC_50_ for inhibition of bacterial type IA topoisomerases (Figure 6a). Moreover, anziaic acid at up to 230 µM did not result in increase in cleavage product formed by human topoisomerase I (Figure 6b).

**Figure 7:**
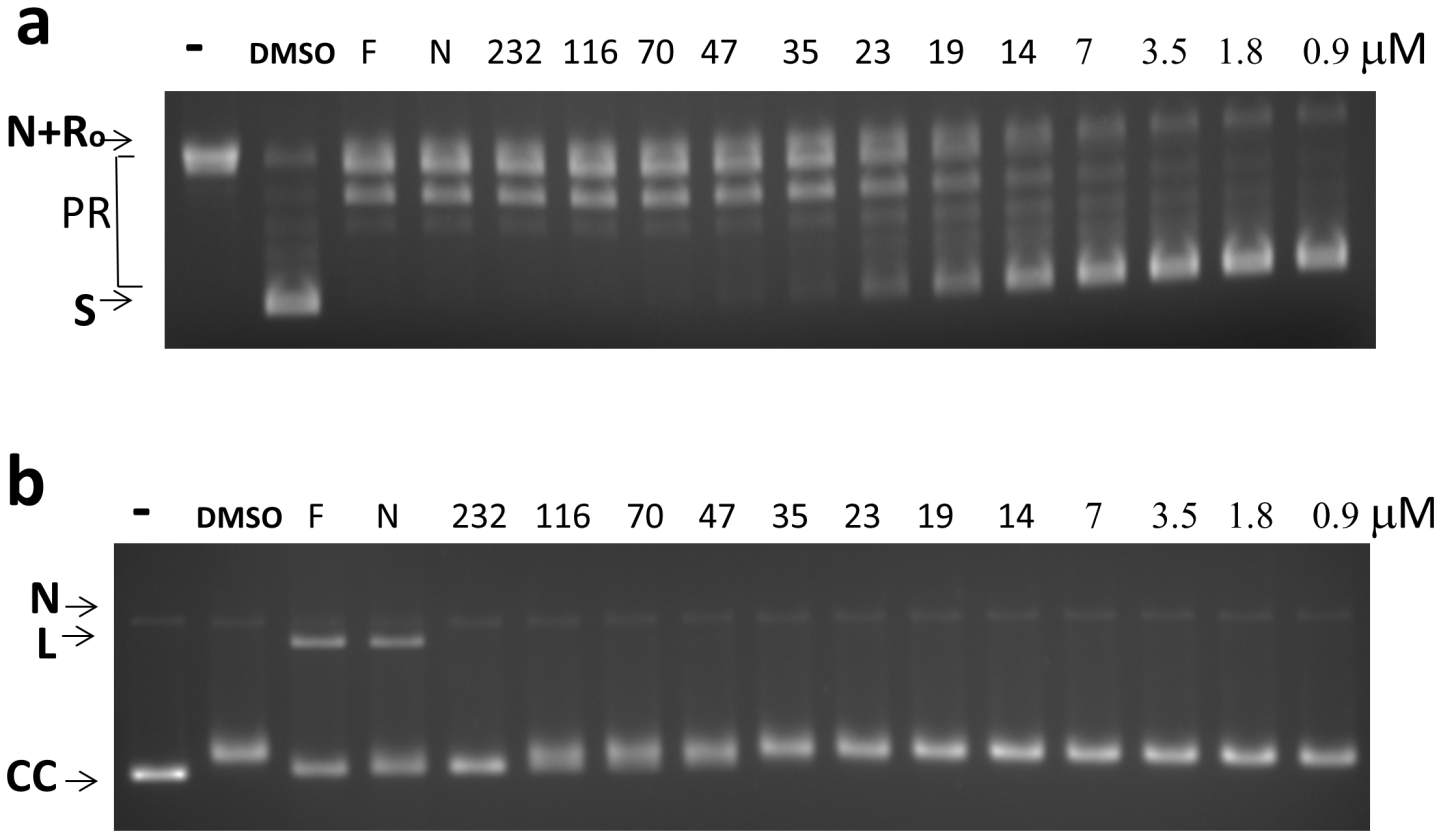
Effect of anziaic acid on *E. coli* DNA gyrase activity. (a) Assay of supercoiling activity with 1 U of enzyme and 250 ng of relaxed plasmid DNA substrate. (b) DNA cleavage assay with 5 U of enzyme. Gel electrophoresis was carried out in the presence of 0.5 µg/mL ethidium bromide. Positive controls – F: 100 µM levofloxacin; N: 100 µM norfloxacin. S: supercoiled plasmid DNA substrate. N: nicked plasmid DNA. Ro: Relaxed closed plasmid DNA. PR: partially relaxed plasmid DNA. L: linear DNA CC: covalently closed circular DNA.

The supercoiling activity of *E. coli* DNA gyrase was assayed using relaxed plasmid DNA substrate along with assay of DNA cleavage product accumulation to determine the effect of anziaic acid on this type IIA DNA topoisomerase. The results showed that while anziaic acid can inhibit the supercoiling activity of DNA gyrase (IC_50_ = 19 µM), it did not act as a poison inhibitor for DNA gyrase over the range of concentrations tested, in contrast to the quinolone antibiotics levofloxacin and norfloxacin (Figure 7).

**Figure 8:**
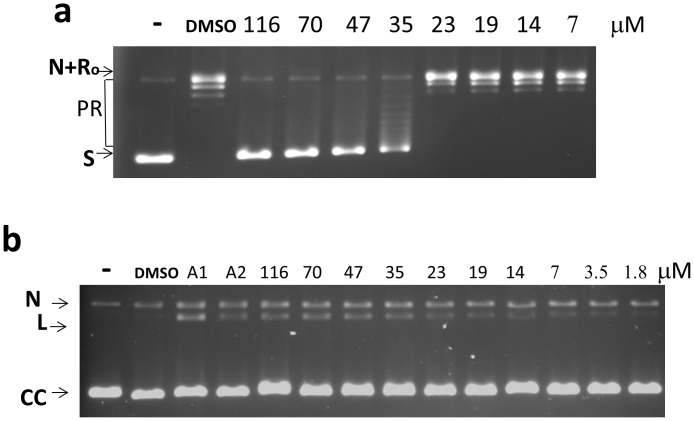
Effect of anziaic acid on human topoisomerase IIα activity. (a) Assay of relaxation activity –1 U of enzyme was used in each relaxation reaction with 250 ng of supercoiled plasmid DNA in the presence of 0.0025% Tween 20. (b) DNA cleavage assay with 5 U of enzyme. Gel electrophoresis was carried out in the presence of 0.5 µg/mL ethidium bromide and m-AMSA was used as a positive control at 25 µM (A1) or 8 µM (A2). -: no enzyme. S: supercoiled plasmid DNA substrate. N: nicked plasmid DNA. Ro: Relaxed closed plasmid DNA. PR: partially relaxed plasmid DNA. L: linear DNA. CC: covalently closed circular DNA.

Human topoisomerase IIα is also a type IIA topoisomerase. Anziaic acid was found not only to be an inhibitor of human topoisomerase IIα relaxation activity (IC_50_ = 35 µM) when assayed with supercoiled plasmid DNA (Figure 8a), but was also found to act as a poison inhibitor for increase of the human topoisomerase IIα linear DNA cleavage intermediate at concentration below this IC_50_ (Figure 8b).

### Cytotoxicity Evaluation

To determine potential cytotoxicity of anziaic acid, human pulmonary artery endothelial cells were treated with different concentrations of anziaic acid. Luciferase linked ATP assay was used to measure viability. When compared to DMSO control, concentration of 21 µM anziaic acid was found to result in fifty percent loss of viability.

## Discussion

Natural products are a valuable resource of diverse chemical structures with potentially useful therapeutic activity. There is an urgent need for novel antibacterial compound due to the increased prevalence of drug resistant pathogens in both hospital and community settings. Bacterial topoisomerase I is a promising new target for antibacterial therapy, but inhibitor prototypes need to be identified to initiate the drug discovery efforts. Topoisomerase poison inhibitors are especially valuable as antibacterial or anticancer drugs because of the active cell killing mechanism that requires only a small percentage of the target topoisomerase molecules be trapped on chromosomal DNA as a result of the poison inhibitor action.

A HTS assay was used to explore a collection of natural product extracts for poison inhibitors targeting *Y. pestis* topoisomerase I, which shares 85% sequence identity with the more extensively characterized *E. coli* topoisomerase I. The two enzymes have shown identical properties in previous biochemical studies [9], [16], [25]. *Y. pestis* topoisomerase I was used in the initial HTS because the NERCE/NSRB resource can be readily available for this biodenfense related target. Two of the primary assay hits corresponded to lichen *Hypotrachyna* sp. extracts. While the extracts likely contained multiple compounds capable of inhibition of the relaxation activity of bacterial topoisomerase I, a unique fraction from the HP-20ss fractionation showed properties expected from a topoisomerase poison inhibitor. Further purification led to the identification of the depside anziaic acid as a bacterial topoisomerase I poison inhibitor. While the ester linkage in anziaic acid could potentially be hydrolyzed, mass spectrometry analysis confirmed the continued presence of the intact compound following incubation in the topoisomerase assay buffer (data not shown). Preliminary SAR studies (results to be published) showed that synthesized compounds with structures identical or similar to the products expected from the ester hydrolysis of anziaic acid did not inhibit bacterial growth or topoisomerase activity, providing support that anziaic acid is the active inhibitor.

Bacterial topoisomerase I enzymes belong to the type IA topoisomerase subfamily. This class of enzyme shares no similarity in sequence or mechanism with the type IB topoisomerase subfamily other than the use of an active site tyrosine nucleophile. Anziaic acid had an extremely weak effect on the human topoisomerase I, a type IB enzyme, indicating that it is not a non-specific inhibitor for all DNA enzymes. It does not act as a promiscuous inhibitor by causing protein aggregation [26] because the presence of detergent Tween 20 had minimal effect on its inhibition potency for bacterial topoisomerase I. The hydroxyl and carboxyl moieties in the structure of anziaic acid point to the possibility of divalent ion chelation as a potential mechanism of inhibition, since divalent ions are required for the religation step of the bacterial topoisomerase I catalytic cycle. However, the potency of bacterial topoisomerase I inhibition did not decrease when the concentration of Mg^2+^ in the reaction shifted from 0.5 mM to 6 mM. Therefore anziaic acid did not inhibit bacterial topoisomerase I by competing for binding of free divalent ions. It could potentially inhibit bacterial topoisomerase I by interacting with the divalent ions bound at the enzyme active site, similar to the mechanism of action of the raltegravir, an inhibitor for the strand transfer step of HIV integrase [27]. Anziaic acid was also found to inhibit the supercoiling activity of *E. coli* DNA gyrase, a type IIA topoisomerase, although it was not found to increase the gyrase DNA cleavage product as a poison inhibitor. Further studies are needed to confirm that topoisomerase I is the antibacterial target for anziaic acid.

Anziaic acid was also found to act as a poison inhibitor against human topoisomerase IIα. This could account at least in part for the cytotoxicity observed for human pulmonary arterial endothelial cells. Human topoisomerase IIα is another member of the type IIA topoisomerase subfamily. Type IA and type IIA topoisomerase subfamilies share a number of structural and mechanistic similarities including interaction with divalent ions with conserved aspartates in the TOPRIM domain in the active site [28], [29]. Mg^2+^ ions are required for DNA rejoining by type IA topoisomerases, but are dispensable for DNA cleavage by this class of topoisomerase. In contrast, divalent ions are required for DNA cleavage by type IIA topoisomerases. The observed effects of anzaizic acid on the various topoisomerase activities (summarized in Table 2) are consistent with the compound potentially interfering with Mg^2+^ ions interaction in the TOPRIM domain as mechanism of action. Such interference would still allow DNA cleavage by type IA topoisomerases but DNA rejoining would be inhibited to result in accumulation of type IA topoisomerase cleavage intermediate. Type IB topoisomerases do not require divalent ions for catalysis, and would therefore be most resistant to inhibition by anziaic acid. It is intriguing that anziaic acid was found to act as a poison inhibitor for human topoisomerase IIα, but inhibits the catalytic activity of DNA gyrase without an increase in DNA cleavage intermediate. The two metals found at the active site of these type IIA enzymes might be affected differentially by anziaic acid, allowing DNA cleavage to take place for human topoisomerase IIα, but not for DNA gyrase.

**Table 2:** Summary of inhibitory action of anziaic acid on topoisomerases tested.

Topoisomerase Target	IC_50_ for Inhibition of catalytic activity	Poison Inhibitor Mechanism
*E. coli* and *Y. pestis* Topo I (Type IA)	14–19 µM	Yes
*E. coli* DNA gyrase Type IIA)	19 µM	No
Human Topo I (Type IB)	230 µM	No
Human Topo Iiα (Type IIA)	35 µM	Yes

Lichens not only might be eaten by animals, but also threatened by antagonistic bacteria, and it is believed that they produce secondary metabolites as defenses against these predators and pathogens [30]. Depsides are common lichen natural products. The antibacterial activity and cytotoxicity of anziaic acid from *Hypotrachyna sp*. provide further evidence that depsides could be lichens’ weapons against animals and microorganisms. Among the ∼200 depsides previously identified from lichens [31], many have antimicrobial activity, including those isolated from *Origanum dictamnus* [32]. Investigation of other natural product depsides or structural analogs of anziaic acid could lead to compounds with greater selectivity towards bacterial topoisomerase I useful for development of new antibacterial agents while similar compounds with greater selectivity for human topoisomerase IIα might have potential as novel anticancer agents. Selectivity for bacterial topoisomerase I and decrease in cytotoxicity could potentially be improved by substituting the lipophilic pentyl group with moieties that can improve the binding affinities to the topoisomerase I protein. This will be followed up in future SAR studies of anziaic acid synthetic analogs.

### Conclusions

A natural product depside was found to be a novel topoisomerase poison inhibitor for bacterial topoisomerase I and human topoisomerase IIα, suggesting the exploratory potential of this class of natural products or analogs as new antibacterial or anticancer agents.

## Supporting Information

Figure S1 **NMR spectra of anziaic acid. (a) ^1^H NMR spectrum. (b) ^13^C NMR spectrum. (c) COSY spectrum. (d) HSQC spectrum. (e) HMBC spectrum.**
(PDF)

Figure S2 **NMR data of anziaic acid.**
(PDF)

Figure S3 **MS spectrum of anziaic acid.**
(PDF)

Table S1 ^13^C and ^1^H spectral data for anziaic acid in CD_3_OD.
(PDF)
