# Supplementary figures and images for: Identification of Anziaic Acid, a Lichen Depside from Hypotrachyna sp., as a New Topoisomerase Poison Inhibitor
Source: PLoS One. 2013 Apr 8;8(4):e60770. doi: 10.1371/journal.pone.0060770 (PMC3620467; doi:10.1371/journal.pone.0060770)

600-6581-4-5  
18 ENERO 2012

Figure S1-a

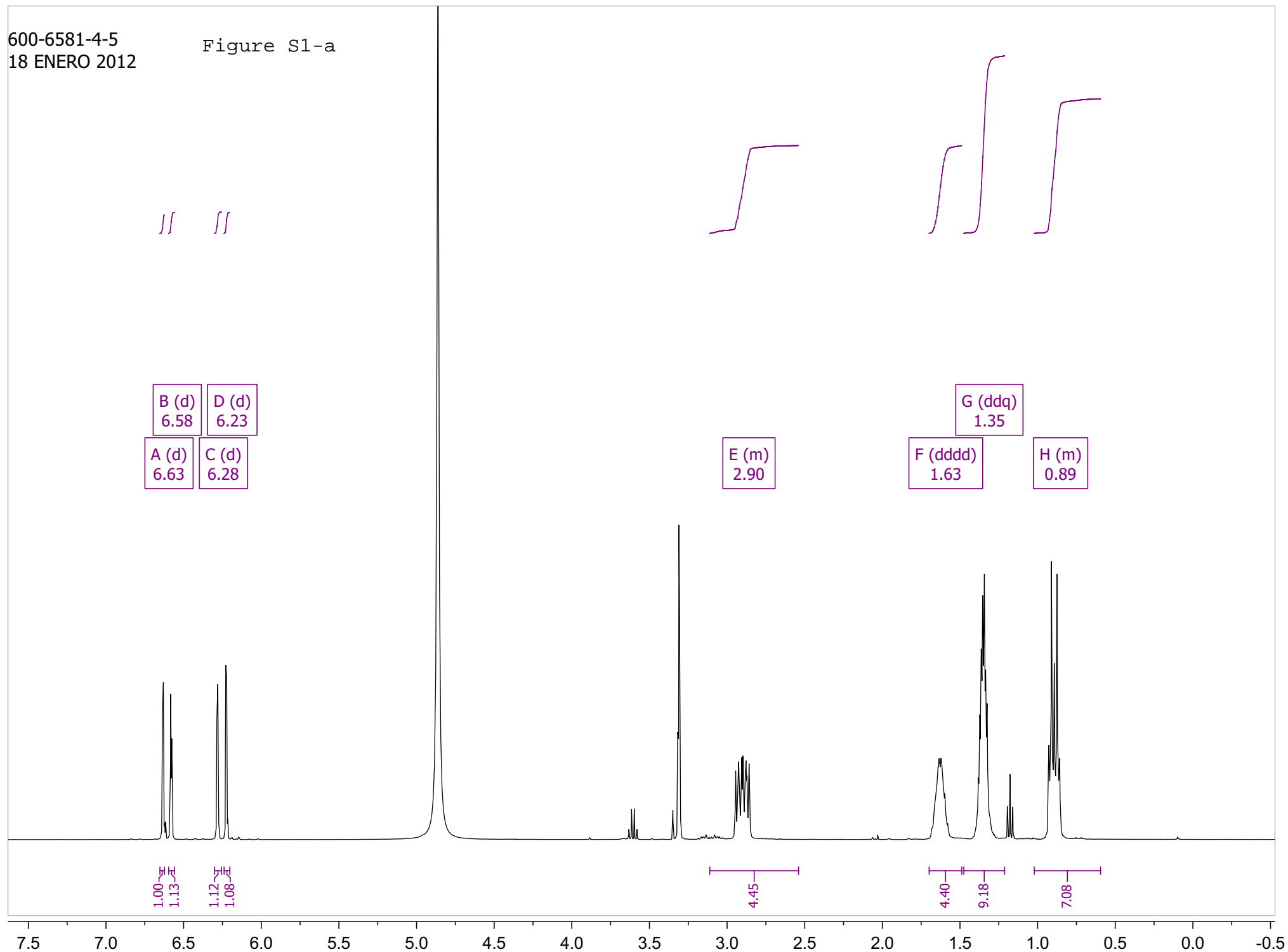

Figure S1-b

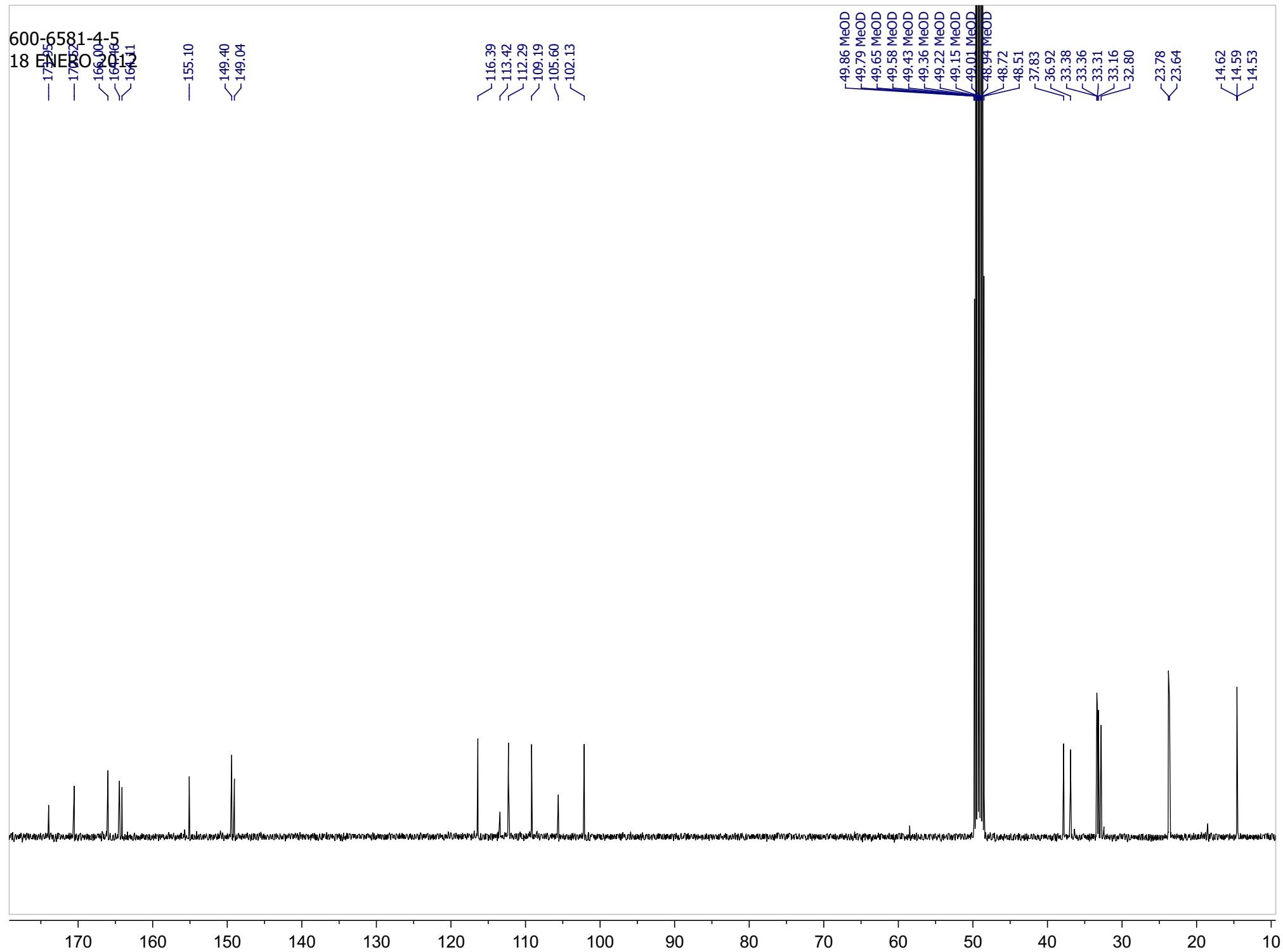

Figure S1-c

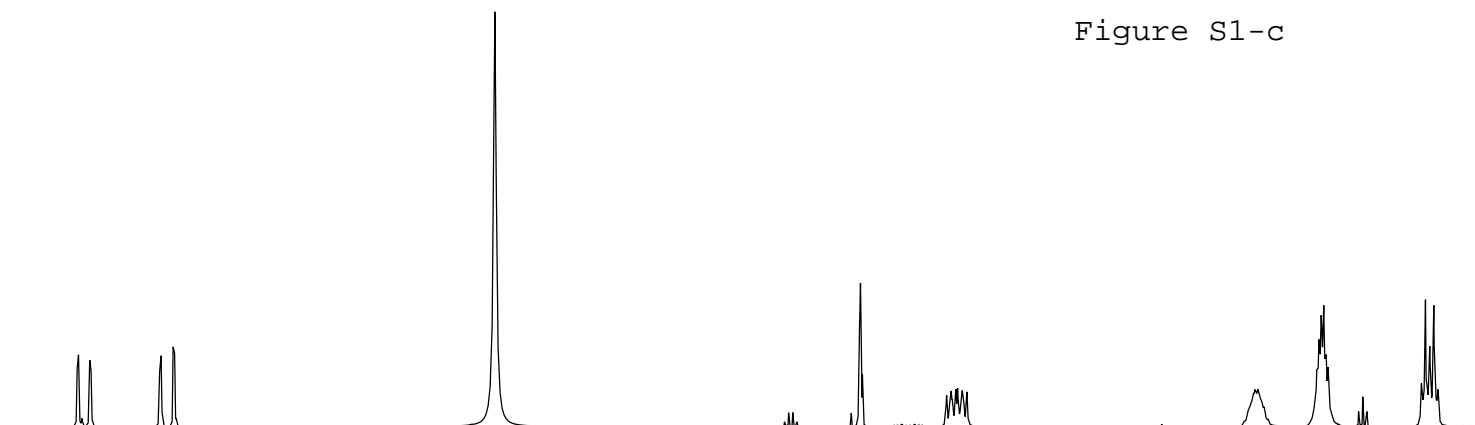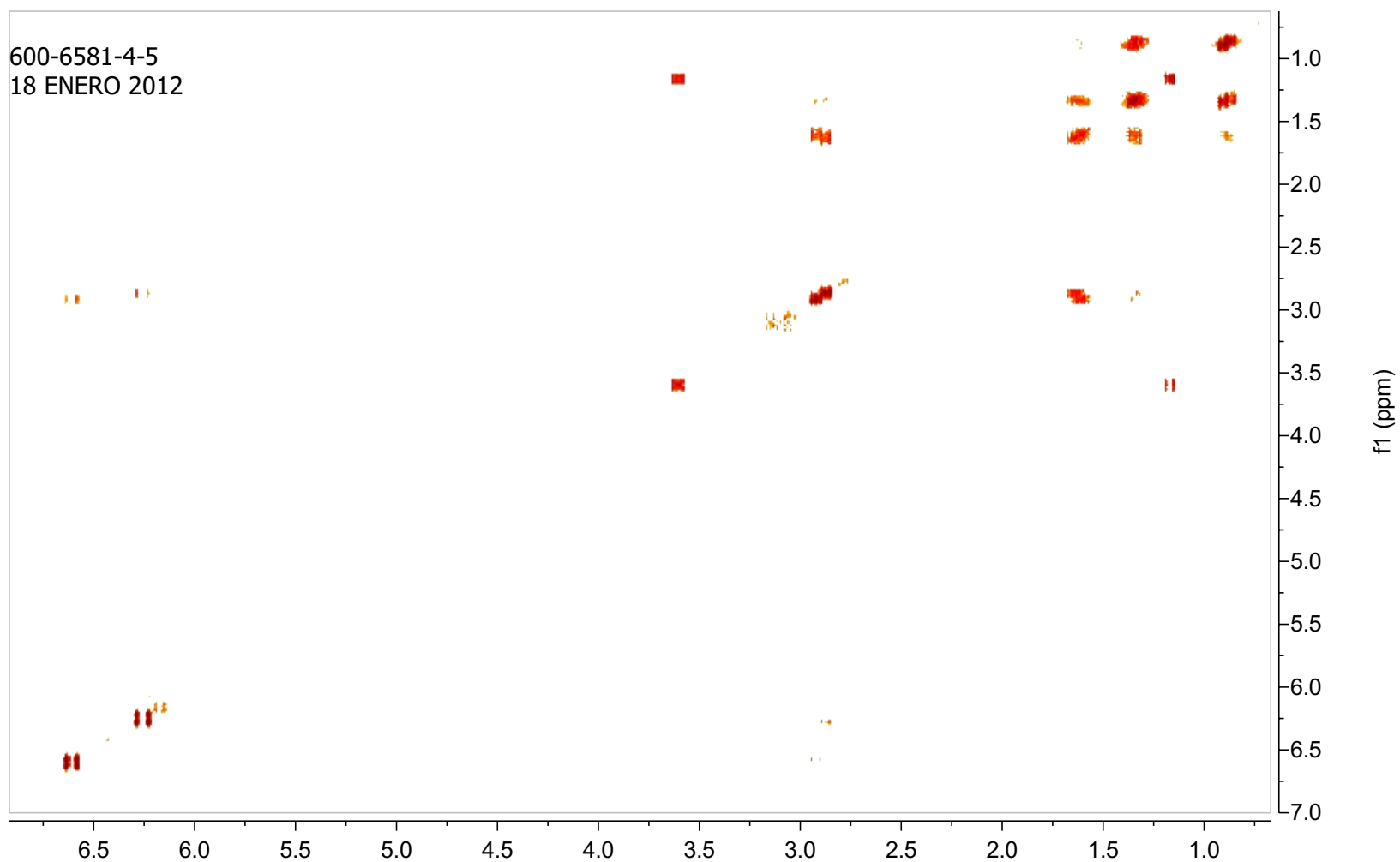

Figure S1-d

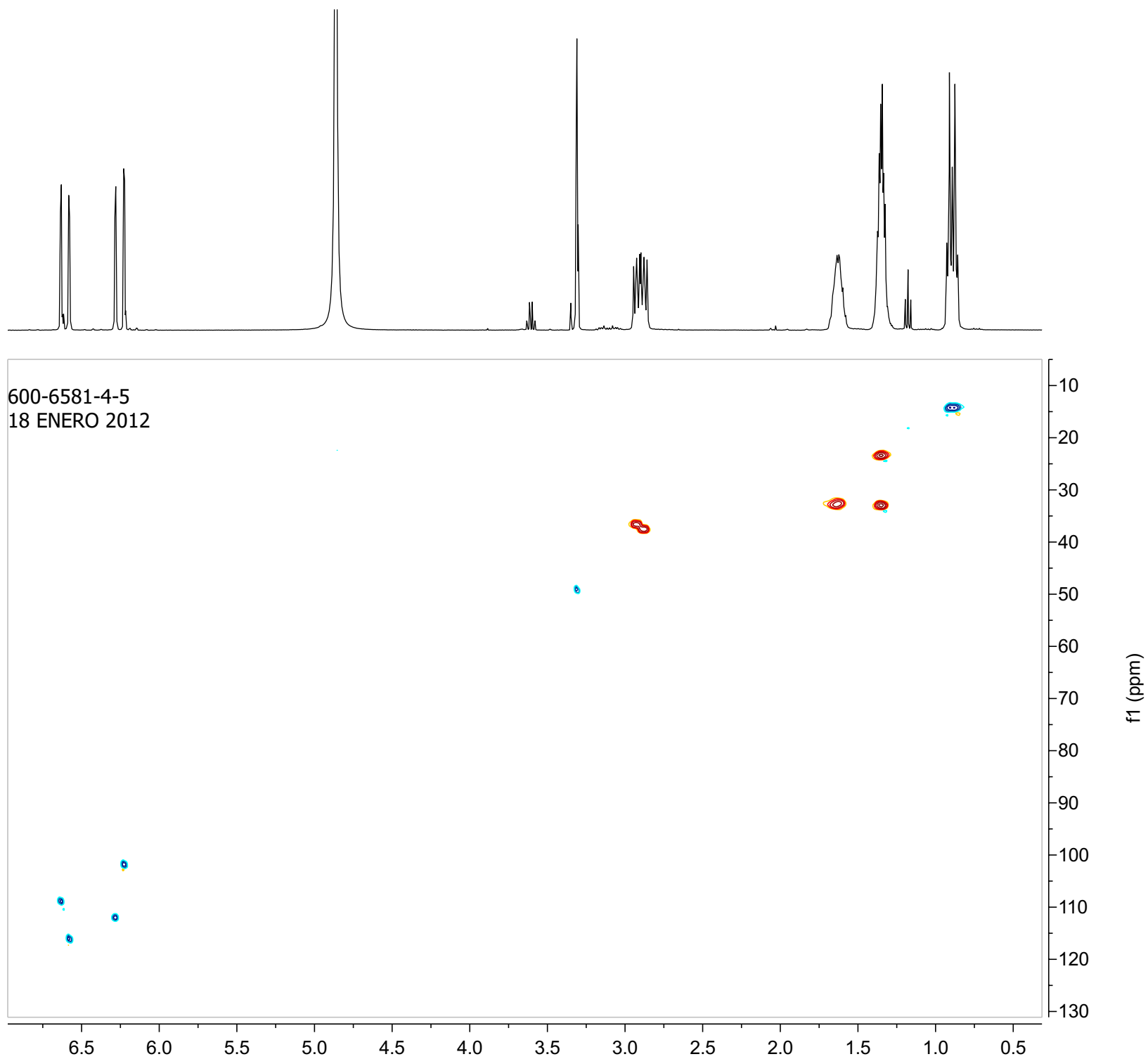

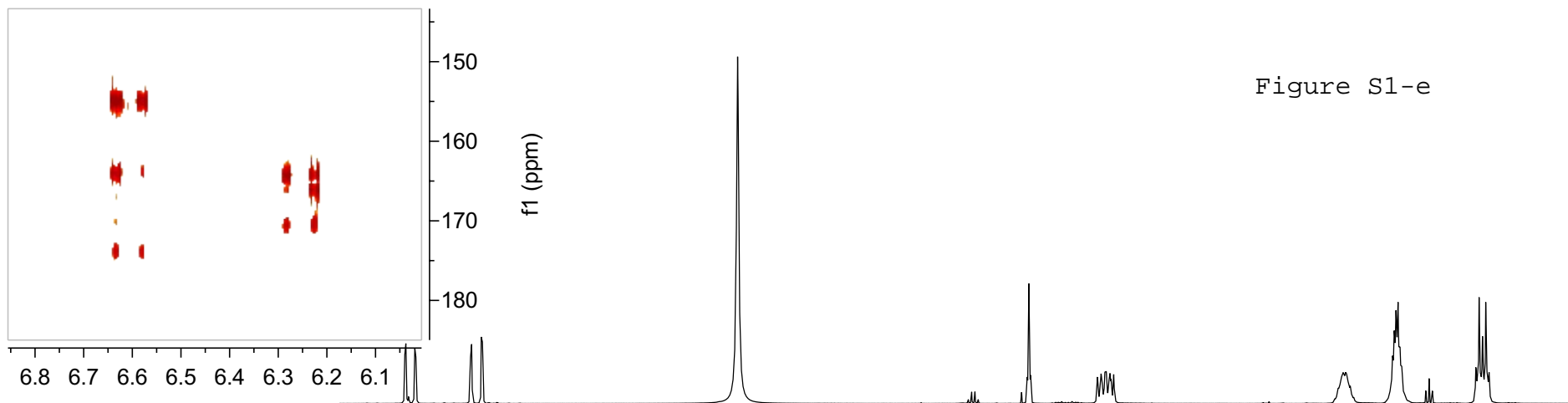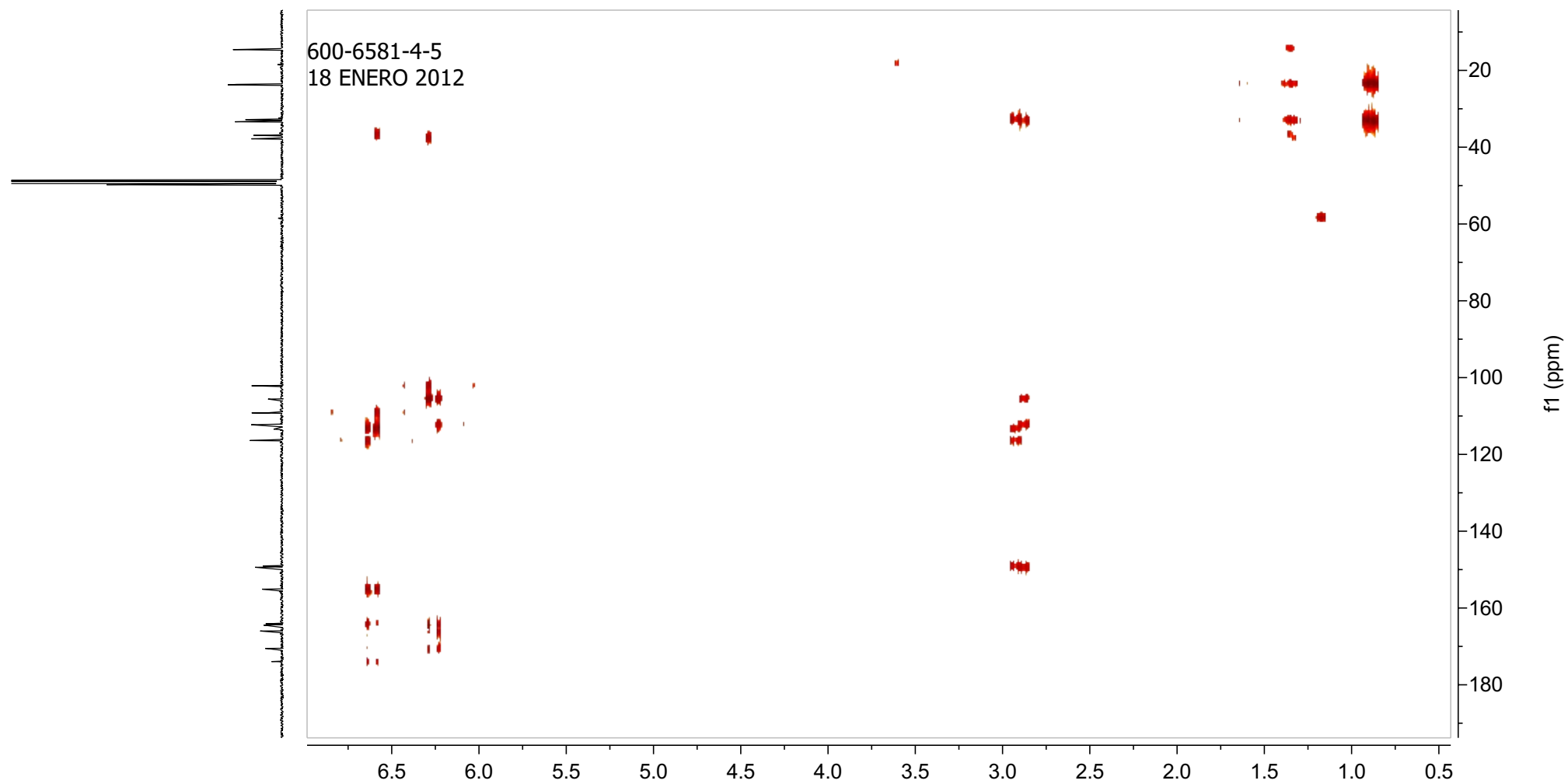

Supplement: Figure S1 — NMR spectra of anziaic acid. (a) 1H NMR spectrum. (b) 13C NMR spectrum. (c) COSY spectrum. (d) HSQC spectrum. (e) HMBC spectrum. (PDF) [file pone.0060770.s001.pdf]

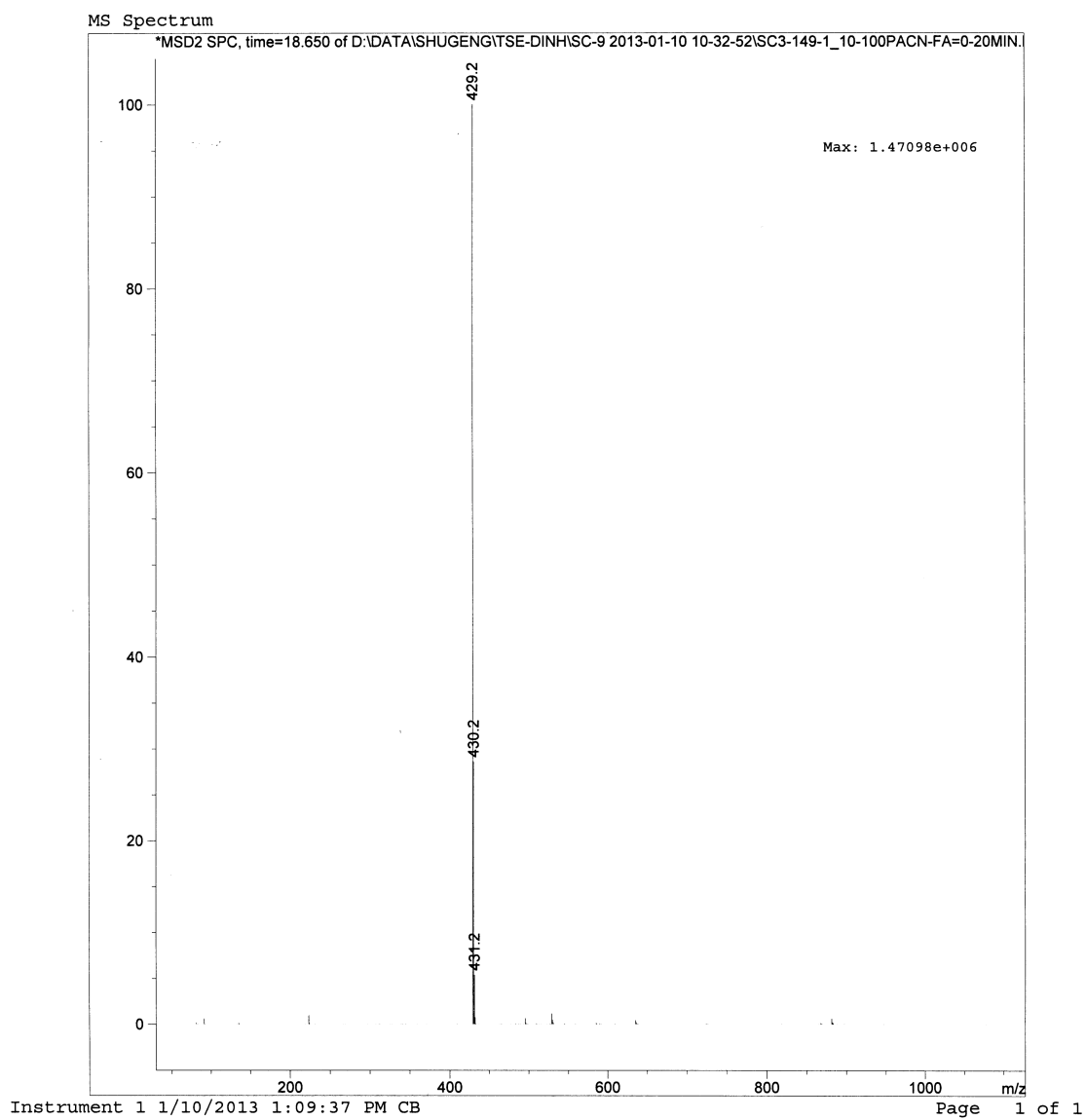

Figure S3

Supplement: Figure S3 — MS spectrum of anziaic acid. (PDF) [file pone.0060770.s003.pdf]
