# Supplementary material for: Identification of Anziaic Acid, a Lichen Depside from Hypotrachyna sp., as a New Topoisomerase Poison Inhibitor
Source: PLoS One. 2013 Apr 8;8(4):e60770. doi: 10.1371/journal.pone.0060770 (PMC3620467; doi:10.1371/journal.pone.0060770)

Figure S2

The $^{1}$H-NMR presents two pair of doublets ($J$=2.42 and 2.32 Hz respectively) in the aromatic region due to two meta-couplings protons at 6.22 and 6.28, and 6.63 and 6.58 δppm. The data presents two rests of $n$-pentyl units and two carbonyls signals attached to the phenyl rings, according to the gHMBC and gCOSY experiments:

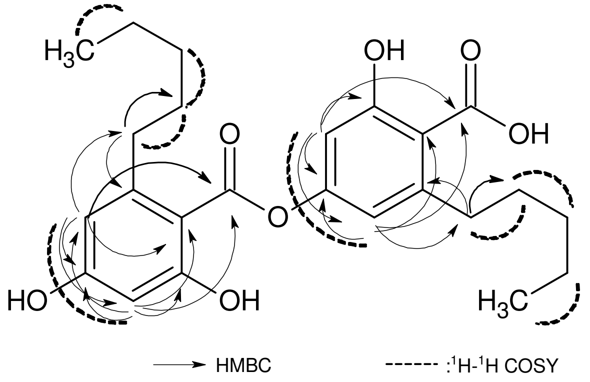

Supplement: Figure S2 — NMR data of anziaic acid. (PDF) [file pone.0060770.s002.pdf]
