# Supplementary material for: Identification of Anziaic Acid, a Lichen Depside from Hypotrachyna sp., as a New Topoisomerase Poison Inhibitor
Source: PLoS One. 2013 Apr 8;8(4):e60770. doi: 10.1371/journal.pone.0060770 (PMC3620467; doi:10.1371/journal.pone.0060770)
Supplement: Table S1 — 13C and 1H spectral data for anziaic acid in CD3OD. (PDF) [file pone.0060770.s004.pdf]

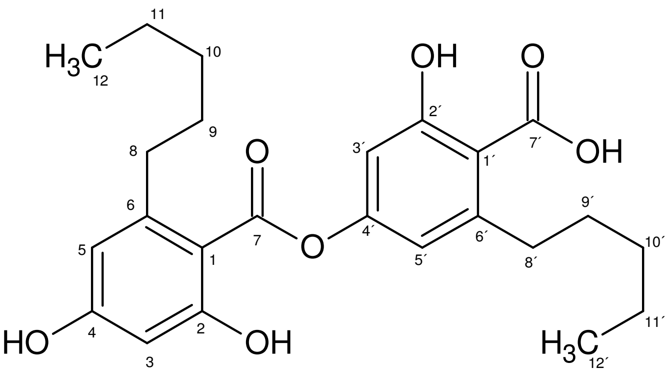

Table S1. ^{13}C and ^{1}H spectral data for anziaic acid in CD_3OD. [a)]

	^{13}C	^{1}H
1	105.60	
2	166.00	
3	102.13	6.22 d (2.42)
4	164.46	
5	112.29	6.28 d (2.42)
6	149.40	
7	170.52	
8	37.83	2.86
9	32.83	1.64
10	32.80 or 33.16 [b)]	1.33
11	23.78 or 23.64 [b)]	1.33
12	14.59 or14.53 [b)]	0.89
1´	113.42	
2´	164.11	
3´	109.19	6.63 d (2.32)
4´	155.10	
5´	116.39	6.58 d (2.32)
6´	149.04	
7´	173.95	
8´	36.92	2.92
9´	32.80	1.64
10´	33.16 or 32.80 [b)]	1.33
11´	23.64 or 23.78 [b)]	1.33
12´	14.53 or 14.59 [b)]	0.89

a) ^{13}C and ^{1}H chemical shifts were recorded at 100 MHz and 400 MHz, respectively.
b) Chemical shifts interchangeable.
